# Post-traumatic Focal Adhesive Arachnoiditis

**DOI:** 10.5334/jbr-btr.1161

**Published:** 2017-02-20

**Authors:** V. Zigic, J. Ristic, I. Turkalj, Filip M. Vanhoenacker

**Affiliations:** 1 Centre of Radiology, Clinical Centre of Vojvodina, Novi Sad, Serbia; 2 University of Novi Sad, Medical Faculty, Novi Sad, Serbia; 3 Department of Radiology, AZ Sint-Maarten Duffel/Mechelen, Mechelen, BE; 4 Department of Radiology, Antwerp University Hospital, Edegem, BE; 5 University of Ghent, Faculty of medicine and Health sciences, Ghent, BE

**Keywords:** trauma, spine, arachnoiditis

A 31-year-old male prisoner and a former drug addict was referred for magnetic resonance imaging (MRI) of the thoracic spine due to longstanding walking difficulties. His complaints started shortly after he was beaten with a baseball bat in the region of his back. On admission, the patient suffered from disturbed walking scheme with bilateral spastic hypertonia and reduced muscle strength of the lower limbs. Further neurological examination was unremarkable, while laboratory findings were within the range of normal limits.

MRI examination showed widespread intradural bands of low T2-signal (Figure 1A–C, arrows) showing vivid enhancement (Figure 1D and E, arrows). There was distortion of the thoracic spinal cord, especially at Th7/8 level where the cord was displaced posteriorly (Figure 2A and B, arrows). At this level, the spinal cord showed marked T2-hyperintense signal in keeping with myelomalacia (Figure 2A–C, arrowheads). There was cord atrophy below the level of Th8 extending to the conus medullaris (Figure 2A, asterisk). Based on imaging and clinical findings, the diagnosis of post-traumatic focal adhesive arachnoiditis was made.

**Figure 1:**
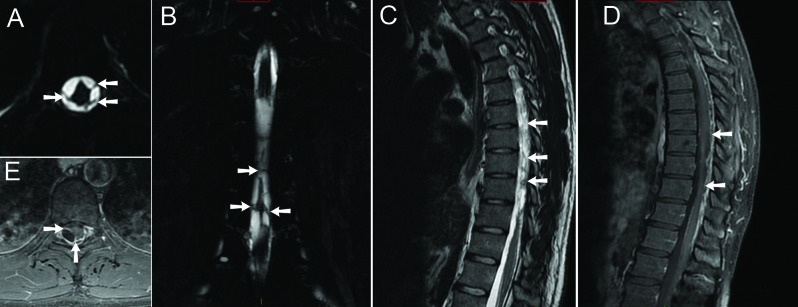
**A.** Axial fatsuppressed (FS) T2-weighted image (WI). **B.** Coronal FS T2-WI. **C.** Sagittal T2-WI. **D.** Sagittal FS T1-WI after administration of intravenous gadolinium contrast. **E.** Axial FS T1-WI after administration of intravenous gadolinium contrast.

**Figure 2:**
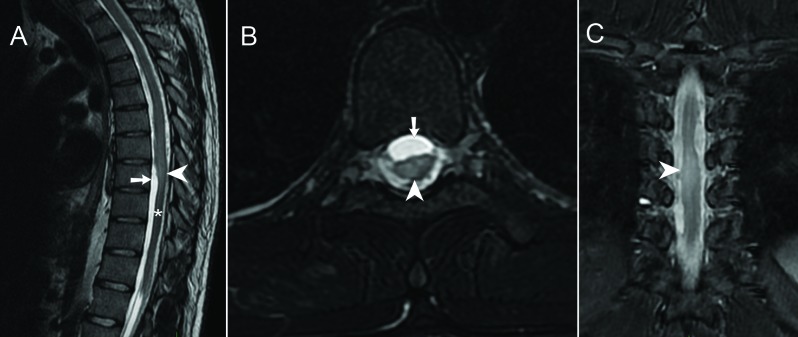
**A.** Sagittal T2-WI. **B.** Axial FS T2-WI. **C.** Coronal FS T2-WI.

## Comment

Post-traumatic focal adhesive arachnoiditis is a non-infectious inflammatory condition caused by previous hemorrhage in the spinal canal as a consequence of excessive stretching of the superficial vessels or mechanical compression from a vertebral fracture. The bleeding may induce inflammatory reaction resulting in formation of adhesions between the spinal cord and dura. This may further cause fixation of the spinal cord and obstruction of spinal cerebrospinal fluid (CSF) circulation. Changed CSF pressure above and below the area of mechanical obstruction could direct CSF into the spinal cord through Virchow-Robin space. This mechanism may explain syrinx formation or development of myelomalacia [1]. Alternatively, vascular compromise by surrounding scars may result in cord ischemia.

Differential diagnosis includes idiopathic spinal cord herniation in which there is absence of scar formations in subarachnoid space, and arteriovenous malformation characterized by serpiginous intradural flow voids originating from aberrant blood vessels.

The treatment comprises of CSF shunting into the peritoneal or pleural space in order to prevent further progression of myelomalacia and syrinx formation. In case of recurrent symptomatology microsurgical dissection of the arachnoid adhesions should be considered.
